# Complexity Evaluation of an Environmental Control and Life-Support System Based on Directed and Undirected Structural Entropy Methods

**DOI:** 10.3390/e23091173

**Published:** 2021-09-07

**Authors:** Kaichun Yang, Chunxin Yang, Han Yang, Chenglong Zhou

**Affiliations:** 1 School of Aeronautical Science and Engineering, Beihang University, Beijing 100191, China; zy1905425@buaa.edu.cn (K.Y.); chunxinyang@buaa.edu.cn (C.Y.); 2 Institute of Microelectronics of the Chinese Academy of Sciences, Beijing 100191, China; zhouchenglong@ime.ac.cn

**Keywords:** environmental control and life-support system, top-level scheme, structural entropy, structural complexity, order degree

## Abstract

During manned space missions, an environmental control and life-support system (ECLSS) is employed to meet the life-supporting requirements of astronauts. The ECLSS is a type of hierarchical system, with subsystem—component—single machines, forming a complex structure. Therefore, system-level conceptual designing and performance evaluation of the ECLSS must be conducted. This study reports the top-level scheme of ECLSS, including the subsystems of atmosphere revitalization, water management, and waste management. We propose two schemes based on the design criteria of improving closure and reducing power consumption. In this study, we use the structural entropy method (SEM) to calculate the system order degree to quantitatively evaluate the ECLSS complexity at the top level. The complexity of the system evaluated by directed SEM and undirected SEM presents different rules. The results show that the change in the system structure caused by the replacement of some single technologies will not have great impact on the overall system complexity. The top-level scheme design and complexity evaluation presented in this study may provide technical support for the development of ECLSS in future manned spaceflights.

## 1. Introduction

Environmental control and life-support systems (ECLSS) are utilized to meet the survival requirements of astronauts in a space environment. ECLSS can provide a habitable environment with a suitable atmosphere, as well as basic necessities such as oxygen, drinking water, and food for astronauts. In addition, it can remove human waste, CO_2_, wastewater, urine, and feces. Moreover, the system can realize the functions of pressure relief protection, fire detection and extinction, and harmful gas removal [1,2,3].

At present, the research of ECLSS is divided into system-level and single-technical-level. System-level research includes overall technical progress [4,5], testing [6,7], software simulation, [8,9] and performance evaluation [10,11]. Single-technical level-research is mainly the design and optimization of specific components, such as carbon dioxide reduction [12], oxygen regeneration [13], water management [14], and waste management [15].

There has been some research on top-level design and evaluation included in system-level study. Top-level design is the overall scheme design formed by system hierarchy and corresponding technology selection, which is crucial for the overall optimization and evaluation of ECLSS. Levri [16,17,18] discussed the metric method and calculation procedure of equivalent system mass, which is often applied to evaluate trade study options in the advanced life-support program. Around 2000, Rodriguez [19], Goudarzi [20] and others [21,22] established the dynamic top-level models of an advanced life-support system, including crew, crop, waste processing and resource recovery, food processing and nutrition. The system performance was further analyzed by coding the models. Based on the life-support technology in 2004, Czupalla [23] designed an overall scheme for a life-support system for the purpose of meeting the top-level requirements of a Mars mission.

With the upgrading of space missions, advanced ECLSS will be further developed [24]. Research on top-level design and evaluation will also receive further attention. Meyer [25] summarized the development status and technology development fields of ECLSS in recent years. Owens [26] comprehensively analyzed the overall impact that different ECLSS architectures have on the quality of the crewed Mars missions and considered the decision-making impact of ECLSS architectures on other mission architectures, such as transportation systems. Cremaschi [27] evaluated the physicochemical life-support system, biological regeneration life-support system and hybrid life-support system to investigate the effects of different technologies on the overall system cost and supply requirements. Based on the water management and atmosphere revitalization subsystem, Eshima [28] studied the system-level fault propagation and found that the interaction with other subsystems affected the entire system.

ECLSS is a nonlinear system with a complex structure, owing to its multiple levels, such as system-subsystem-component-single machine, and different levels possess coupling relationships between material flow and energy transfer. Because every technology has various alternative approaches, many types of technology combinations may exist [29]. Although the ECLSS of the international space station (ISS) has achieved a very challenging goal, it has not adopted a standby system architecture at the system level, and its technical reliability has not been quantitatively analyzed. Owing to the lack of an efficient design, there are some specific problems in its operation, such as high power consumption, difficult maintainability, and the sensitivity of several components to particulates and fouling [30]. Thus, insufficient system-level design and quantitative analysis for such a complex system can lead to inaccurate estimation of the actual overall performance of the system and may cause unforeseen performance loss.

The quantitative evaluation of ECLSS top-level scheme can adopt many different evaluation indices, such as equivalent system mass [17], closure degree [31], complexity [31], robustness [32], life-cycle cost [33], etc. The complexity of ECLSS has a considerable impact on the physical strength of astronauts, because it increases the control difficulty and labor burden on the astronauts. For example, the switch operation of electrolyzed water and the Sabatier reactor on ISS must be performed by astronauts as per instructions from ground communication [30,31]. Moreover, systems with higher complexity generally have higher costs and failure rates [34]. How to quantitatively evaluate complexity is one of the key points in ECLSS design. In 2020, Jones first proposed a system complexity metric (SCM) for ECLSS, which is used to predict costs and failure rates [34]. Then, the SCM was used to evaluate the complexity of a carbon dioxide reduction system [35]. The SCM measures complexity by calculating the number of single machines or connections in the actual physical system structure, which is suitable for the initial screening of ECLSS technology [34]. If the SCM is not sufficiently different, further analysis is needed [35]. However, this calculation method focuses only on the number of system structure nodes and one-way interactions, which ignores the details of the structure or relationship. The top-level scheme evaluation of ECLSS needs to be further investigated.

Since Shannon introduced the theory of information entropy in 1948 [36], it has been widely used in complexity measurement [37,38,39,40,41]. Entropy is also used to quantitatively describe the uncertainty and order degree of system structure, which can facilitate complexity evaluation [42,43,44,45]. Wang [46] built an evaluation model based on information entropy and investigated the order degree of the organizational structure of power regulatory agencies in terms of timeliness and quality of information flow. Yang [47] used information entropy to evaluate the order degree of air cycle systems with different architectures in the cockpit of aircraft. Aziz [48] characterized the complexity of different network graph structures based on information entropy. Existing studies have preliminarily proved that information entropy can be used to evaluate the complexity of system structure.

This study introduces a new way to analyze the ECLSS from the perspective of an information system, describe the top-level structure of an ECLSS through graph-based theory [49], and evaluate complexity using information entropy [39]. To evaluate the complexity of an ECLSS based on the information entropy, we design two kinds of ECLSSs and implemented the structural entropy method (SEM), particularly the undirected structural entropy method (U-SEM) and directed structural entropy method (D-SEM), with different system structures. The contribution of this study is to estimate the complexity of ECLSS based on information entropy theory and propose a calculation method for a top-level evaluation indicator. This study may provide a technical support and analysis method for top-level scheme research into ECLSS in the future.

## 2. Methods

According to the SEM, information transmission in a system network includes the deterministic measurement of timeliness and quality, which represents the efficiency and accuracy of information transmission, respectively [46,47].

### 2.1. Undirected Structural Entropy Method

**Figure 1:**
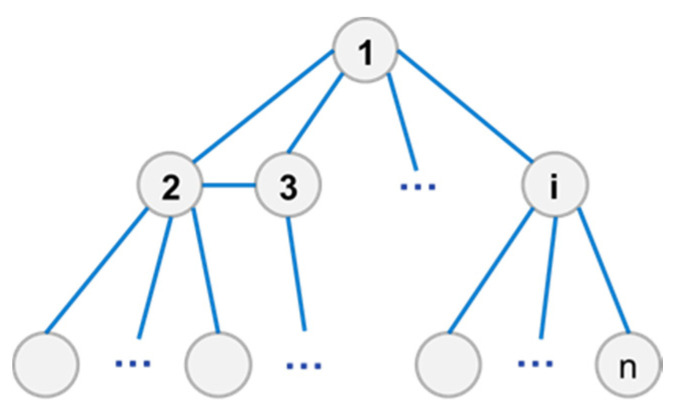
System structure network diagram.

As shown in Figure 1, the elements are abstracted as nodes and the relations are abstracted as edges. All nodes and edges constitute the structural network of the system, between the upper and lower levels, as well as horizontal information relations.

To calculate the structural entropy, the microstate and realization probability of the system must be determined. The microstate of the system represents the quantitative state of the elements when observing the system from one aspect, and the probability of realization is the ratio of the number of microstates of the elements to the sum of all microstates.

The timeliness entropy reflects the uncertainty of the timeliness of information transmission. The shortest distance between any two elements *i* and *j* is known as the timeliness microstate. Timeliness entropy is defined as

(1) $$H_{1}(i,j)=-p_{1}(i,j)\log_{2}p_{1}(i,j)$$

where *p*_1_(*i*, *j*) denotes the realization probability of the timeliness microstates between the *i* and *j* elements of the system (*i*, *j* = 1, 2, 3, …, N).

(2) $$p_{1}(i,j)=\frac{L_{i,j}}{N_{1}}$$

where *L_i,j_* is the minimum channel lengths needed to connect elements *i* and *j* in the system. The length of a directly connected channel is defined as 1, and each information transfer increases the length L by 1. *N*_1_ represents the total number of timeliness microstates.

(3) $$N_{1}=\sum_{i}\sum_{j}L_{i,j}$$

The maximum timeliness entropy of the system is

(4) $$H_{1}{}_{m}=\log_{2}N_{1}$$

The total timeliness entropy of the system is

(5) $$H_{1}=\sum_{i}\sum_{j}H_{1}(i,j)$$

The order degree of the system can be expressed by structure entropy [43,44,47]. Here, the timeliness order degree of the system is defined as

(6) $$R_{1}=1-\frac{H_{1}}{H_{1m}}$$

The quality entropy represents the uncertainty in the quality of information transmission. The microstate of quality is the number of elements directly connected to one element in the system. The quality entropy is expressed as

(7) $$H_{2}(i)=-p_{2}(i)\log_{2}p_{2}(i)$$

where *p*_2_(*i*) denotes the realization probability of quality microstate of system element *i* (*i* = 1, 2, 3, …, N).

(8) $$p_{2}(i)=\frac{K_{i}}{N_{2}}$$

where *K_i_* denotes the number of elements directly connected to element *i* in the system. *N*_2_ denotes the total number of quality microstates.

(9) $$N_{2}=\sum_{i}K_{i}$$

Equations (10)–(12) represent the maximum quality entropy, total quality entropy and quality order degree of the system, respectively.

(10) $$H_{2}{}_{m}=\log_{2}N_{2}$$

(11) $$H_{2}=\sum_{i}H_{2}(i)$$

(12) $$R_{2}=1-\frac{H_{2}}{H_{2m}}$$

The comprehensive order degree *R* of the system is expressed as

(13) $$R=\alpha R_{1}+\beta R_{2}$$

where *α* and *β* are the weights of timeliness and quality, respectively, and *α* + *β* = 1. The larger the value of *R*, the lower complexity of the system structure.

The U-SEM considers unidirectional relationships among the elements of the organizational structure. Additionally, we focus on directionality and establish D-SEM to evaluate the complexity of the system structure.

### 2.2. Directed Structural Entropy Model

**Figure 2:**
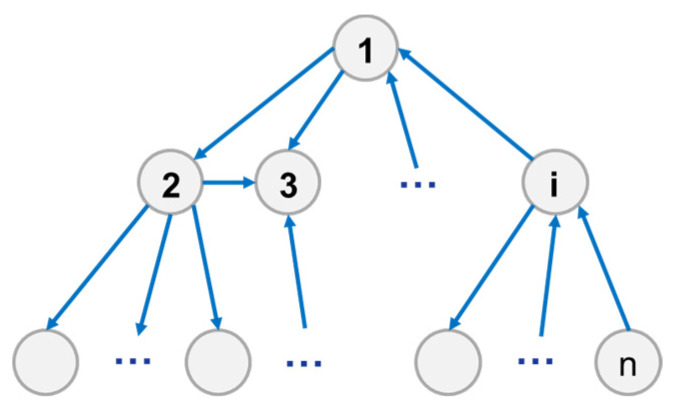
System directed network structure diagram.

Figure 2 shows the network diagram of the system structure, with the arrows indicating the direction of information transmission.

The introduction of the information transfer direction can be regarded as the D-SEM. The timeliness microstate is the shortest path between any two elements *i* and *j*, provided that the path must follow the transfer direction.

(14) $$H_{l}(i\vec{j})=-p_{1}(i\vec{j})\log_{2}p_{1}(i\vec{j})$$

where $p_{1}(i\vec{j})$ represents the realization probability of the timeliness microstate of the element *i* pointing to *j*.

(15) $$p_{1}(i\vec{j})=\frac{L_{i\vec{j}}}{N_{1}}$$

where $L_{i\vec{j}}$ represents the minimum path length required for element *i* to follow the transfer direction towards *j* in the system.

**Figure 3:**
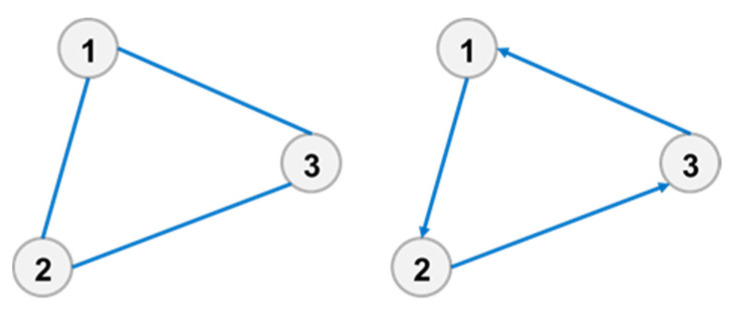
Difference in the timeliness microstate.

For a directional relationship, the calculation results of a timeliness microstate exhibit significant differences, as shown in Figure 3. In the left figure, the shortest distance between element 1 and element 3 is 1. However, in the right figure, the shortest path from element 1 to 3 is 2, and from element 3 to 1 is 1.

Considering the information transfer direction, the quality microstate must be modified according to the number of information transfer directly connected with element *i* in the system. The input and output are calculated separately.

(16) $$H_{2}(\overset{↔}{i})=-p_{2}(\overset{↔}{i})\log_{2}p_{2}(\overset{↔}{i})$$

where $p_{2}(\overset{↔}{i})$ is the realization probability of the quality microstate of the system element *i* considering the inflow and outflow.

(17) $$p_{2}(\overset{↔}{i})=\frac{K_{\overset{↔}{i}}}{N_{2}}$$

where $K_{\overset{↔}{i}}$ denotes the number of information transmission directly connected with element *i* in the system.

**Figure 4:**
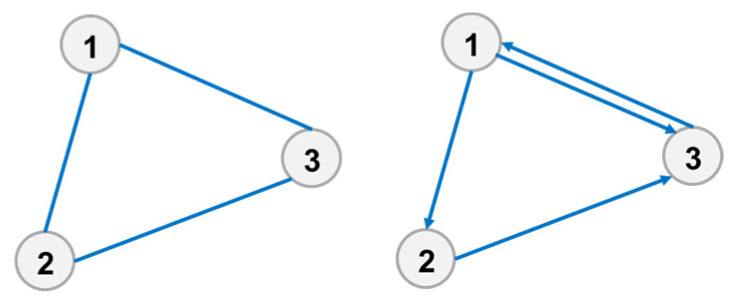
Difference of the quality microstate.

The calculation results of the quality microstate also show significant differences for a directional connection as well, as shown in Figure 4. The quality microstate of element 1, 2, 3 in the left figure is 2, and those of element 1,2,3 in the right figures are 3, 2, and 3, respectively.

Each component of the ECLSS is composed of a complex material, as well as information transmission, and the entire system represents the characteristics of orderly structure and function. With increases in the structure entropy of the system, the complexity and control difficulty of the system increase. Conversely, a smaller structure entropy corresponds to lower complexity and control difficulty.

### 2.3. Uncertainty Analysis

The uncertainty of the results obtained by this method may originate from two aspects: one is the uncertainty of structure, and the other is the uncertainty of information.

The uncertainty of the structure is mainly from the details of the system structure or the identification error of the main components [35]. But the existing structural entropy model has no quantitative method for the uncertainty of the results [46,47]. In addition, there are many other fields of complexity evaluation based on information entropy, such as a stock network [50], a brain network [51], the spatial and temporal entropy of a football game [52], and species distribution [53]. The uncertainty of their calculation results originates from the uncertainty of test data, that was, the uncertainty of information.

The current method considers the timeliness and quality microstate of information in the process of transmission. As long as the system structure is determined, the calculated entropy and order degree reflect the uncertainty and complexity of the structure. However, the current method does not introduce the actual physical system parameters, that is, it does not consider the uncertainty of information, so there is no uncertainty in the current calculation results.

## 3. Top-Level Design

### 3.1. Design Criterion

In the future, the mission scope of manned spacecraft will extend from low Earth orbit to long-lived deep space explorations, such as the moon and Mars expeditions [54]. Owing to the difficulty of replenishment and high cost, the material closure of ECLSS is required to be extremely high or even completely closed.

The life-support system can be divided into two forms: open-loop direct supply and closed-loop recycle regeneration [55]. Open-loop direct supply means to provide O_2_, water and food directly. Closed-loop recycling includes the recovery of all life-support material—oxygen, water, food and other supplies for the crew [56,57]. The ISS partially recovers oxygen and water and conducts food production experiments based on a hybrid life-support system [58,59]. The cost of the consumption mass can be reduced by improving the system closure [55].

This study focuses on the development and evaluation of a physicochemical regenerable life-support system for medium- and long-term missions. Accordingly, the design criteria to reduce the weight cost involve improving system closure and reducing system power consumption.

### 3.2. Scheme

Assuming that several astronauts are on long-term missions on the low Earth orbit space station, we designed an ECLSS scheme I for improving system closure and an ECLSS scheme II for reducing system power consumption. Table 1 presents a comparison of the schemes.

The introduced function of each subsystem and the performance index comparison of different technologies are shown in Supplementary Materials. The closure of top-level scheme I is higher than that of scheme II and the ISS, whereas the power consumption of scheme II is lower than that of scheme I and the ISS.

### 3.3. System Structure

**Figure 5:**
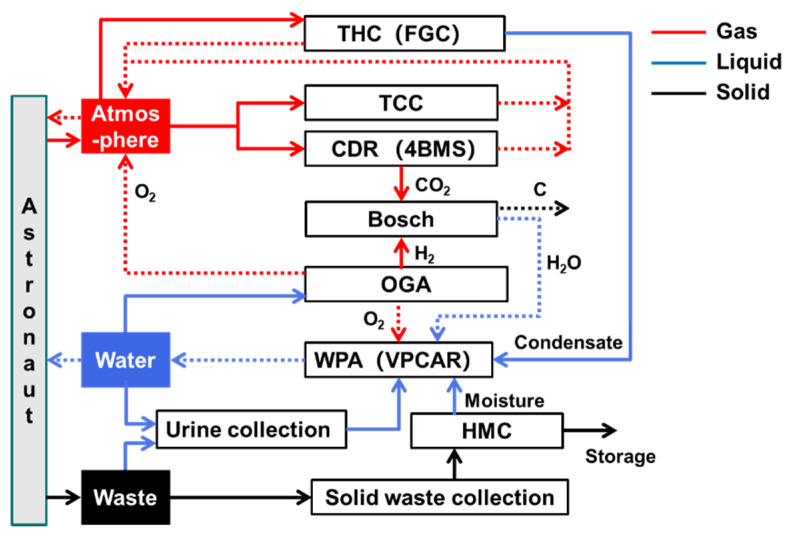
Top-level scheme I.

Figure 5 depicts the subsystem components of top-level scheme I and material transfer relationships among the components.

The astronauts exchange gases with the cockpit atmosphere, i.e., oxygen supply and carbon dioxide exhalation. The atmosphere revitalization subsystem conducts CO_2_ removal, trace contaminant control (TCC) and temperature and humidity control (THC) for the cabin atmosphere. A four-bed molecular sieve (4BMS) is used for carbon dioxide removal (CDR); adsorption and catalytic oxidation are used for TCC, and the first-generation condensation (FGC) module is used for THC. The removed CO_2_ is fed into the Bosch recovery module for oxygen reduction. The oxygen generator assembly (OGA) uses electrolytic water to produce oxygen. The byproduct of oxygen production, hydrogen, is then supplied to the Bosch reactor as a reactant, and all of the treated gases are sent back to the cabin atmosphere.

The water management subsystem can provide drinking water and sanitary water, as well as electrolytic water for oxygen production. Vapor phase catalytic ammonia removal (VPCAR) is applied to the water processing assembly (WPA). The water sources include CO_2_ reduction effluent, condensed water in the cabin atmosphere, urine flushing water and solid waste treatment effluent. Simultaneously, some oxygen is used for catalysis in water treatment.

The waste management subsystem collects urine and solid waste generated by astronauts. Urine washing water is directly passed into the WPA. After the solid waste is treated by the heat melt compactor (HMC), the moisture in the waste is further recovered, and the remaining is stored for treatment.

**Figure 6:**
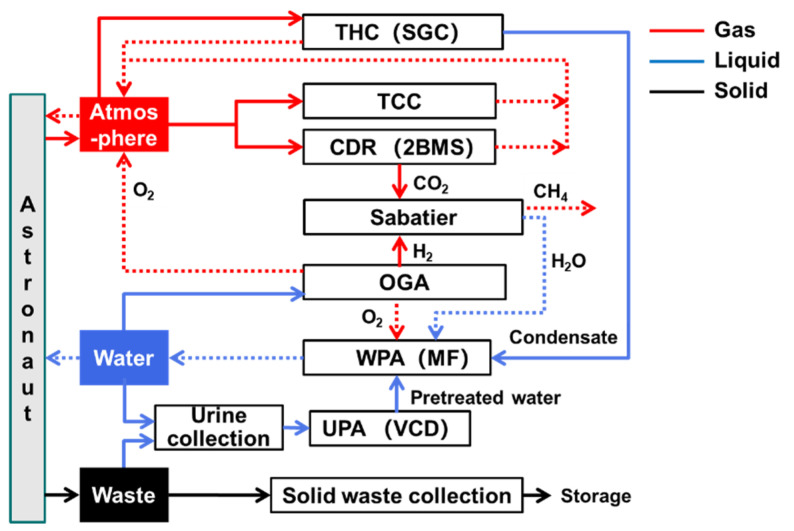
Top-level scheme II.

Figure 6 illustrates the top-level scheme II of the life-support system. Except for different technological choices, the components of each subsystem of both the schemes are similar, and the material transfer between the components is also roughly similar. In the atmosphere revitalization subsystem, two-bed molecular sieves (2BMS) were used for CO_2_ removal; adsorption and catalytic oxidation were used for TCC, and the second-generation condensation (SGC) module was applied for THC. Hydrogen, obtained as a byproduct of oxygen production, was supplied to a Sabatier reactor for CO_2_ reduction. The water is treated using multiple filtration (MF) and vapor compression distillation (VCD). After urine is collected, it is pretreated in the urine processor assembly (UPA) and then passed into the water processing assembly. After solid waste is collected, it can be compressed and stored.

**Figure 7:**
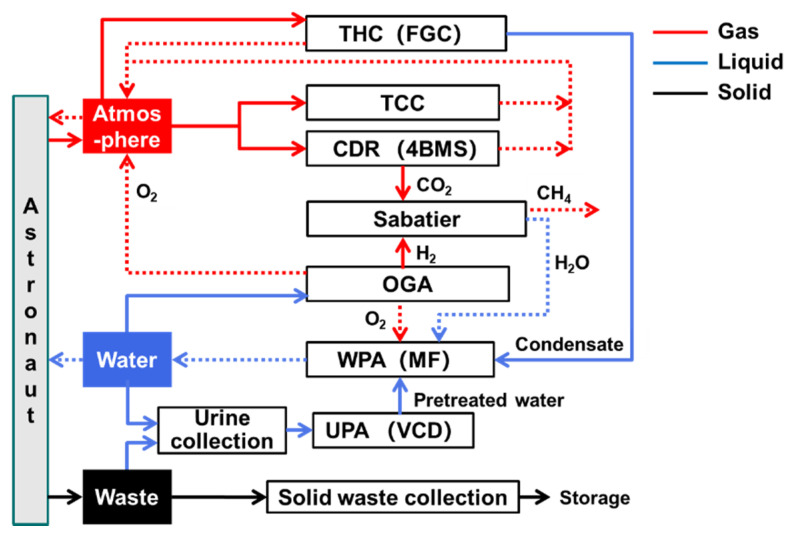
ISS-ECLSS.

Figure 7 presents the structure of the ISS ECLSS. The structure and material transfer relationship is similar to that of the top-level scheme II, except the temperature and humidity control and CO_2_ removal technologies.

## 4. Results and Discussion

### 4.1. Undirected Structural Complexity

**Figure 8:**
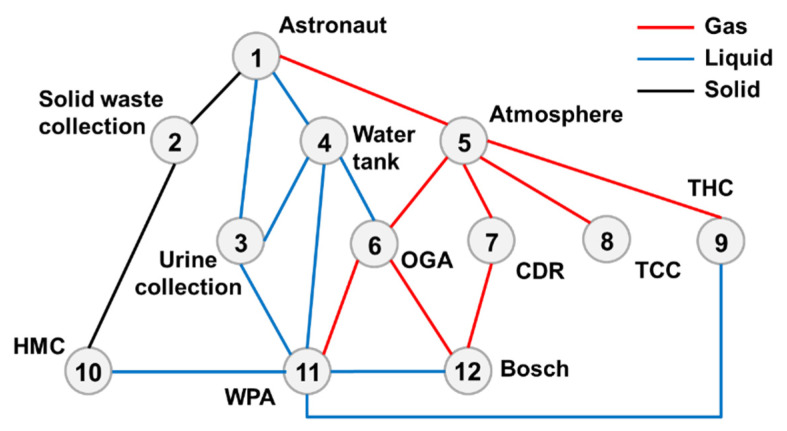
Network diagram of top-level scheme I.

**Figure 9:**
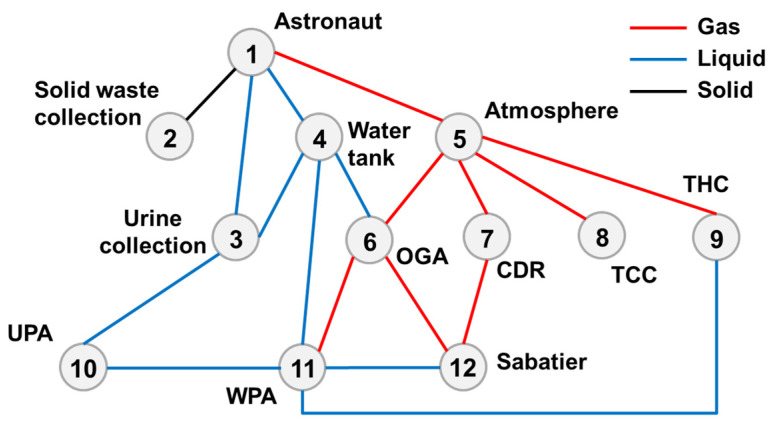
Network diagram of top-level scheme II/ISS.

**Figure 10:**
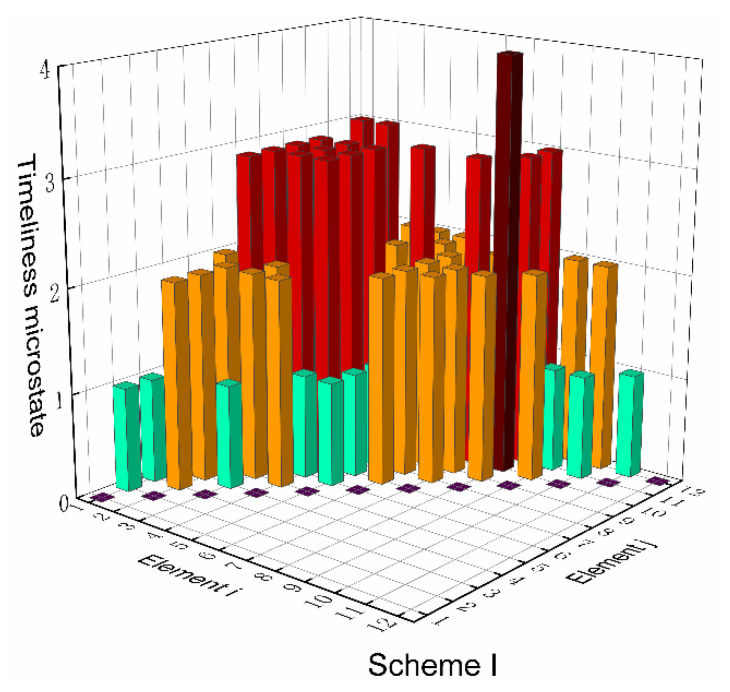
Timeliness microstate distribution of scheme I.

**Figure 11:**
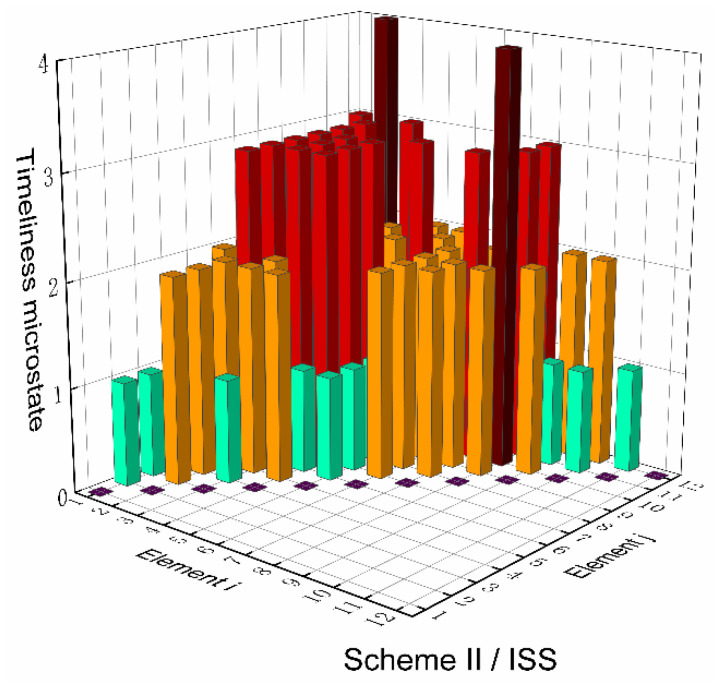
Timeliness microstate distribution of scheme II/ISS.

**Figure 12:**
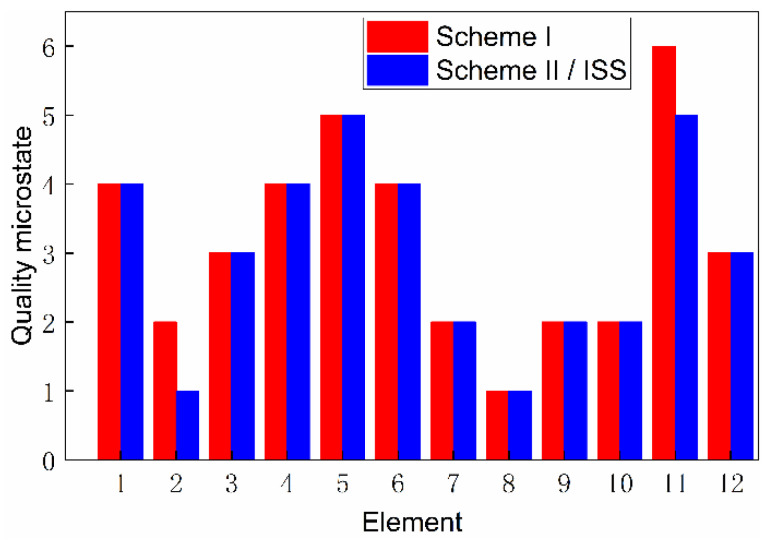
Quality microstate distribution.

Based on the top-level scheme design of the ECLSS, each single machine in the system is regarded as a node, and then the logistics diagram of the system can be abstracted into network. The network diagrams of top-level schemes I and II and the ISS are shown in Figure 8 and Figure 9. According to the network diagram of the top-level scheme, the timeliness microstates and quality microstates of each element are calculated, as shown in Figure 10, Figure 10, Figure 11 and Figure 12.

**Figure 13:**
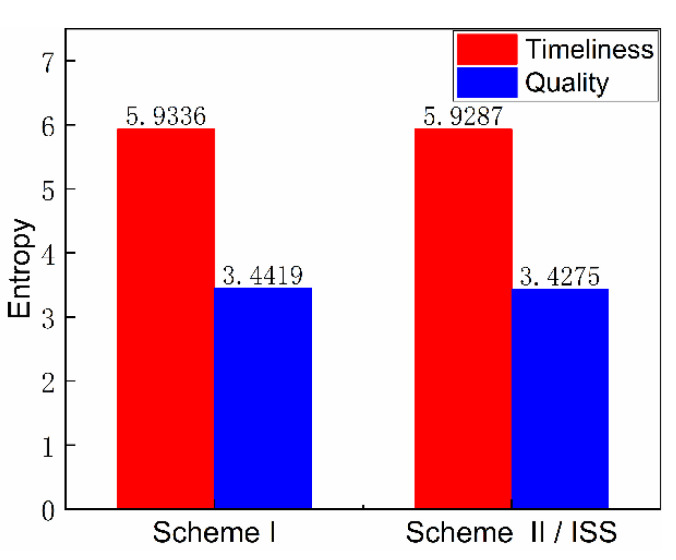
Timeliness and quality entropy.

**Figure 14:**
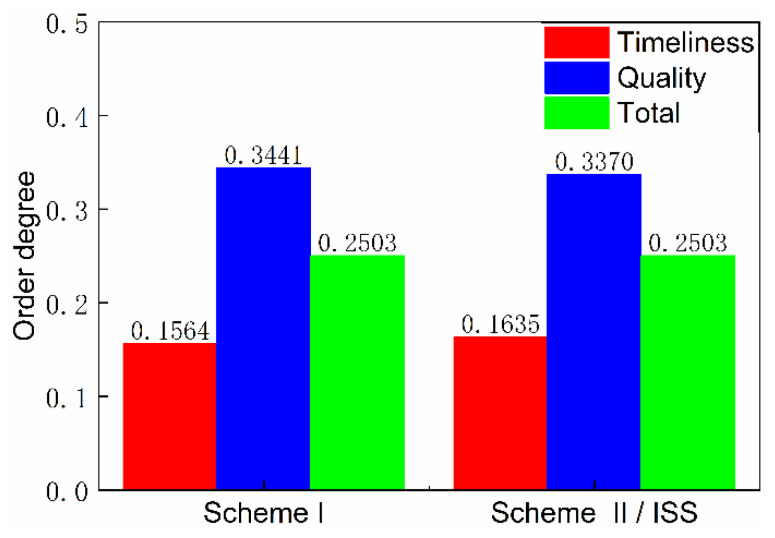
Order degree.

The timeliness entropy and quality entropy of the scheme were obtained by further calculation and statistical processing, respectively, and the order degree was obtained, as shown in Figure 13 and Figure 14. The timeliness and quality entropy of scheme I are both higher than that of scheme II/ISS; the timeliness order degree of scheme I is lower than that of scheme II/ISS. However, the quality order degree of scheme I is higher than that of scheme II/ISS, indicating that it has better information transmission accuracy than scheme II/ISS. Additionally, the transmission efficiency of scheme I is lower than that of scheme II/ISS.

The total order degree is calculated by setting the weight of timeliness and quality order degree to 0.5, respectively. The total order degree of scheme I is equal to that of scheme II/ISS. From the perspective of system network structure, scheme I cancels urine pretreatment due to the centralized water treatment design and adopts HMC technology to recover 25% of the water contained in solid waste, which improves system closure. Although these structure changes cause small differences in timeliness and quality order degree respectively, there is no significant difference in the total order degree.

The numerical difference reflected in the calculation results of undirected structural entropy is extremely small or indistinguishable, which also indicates that the replacement of single-machine technology in the system has little impact on the complexity of the system structure.

### 4.2. Directed Structural Complexity

The material flow direction between the actual single machines is established as per the network diagram of the top-level scheme structure. Therefore, the system complexity is evaluated in terms of dynamic operation.

**Figure 15:**
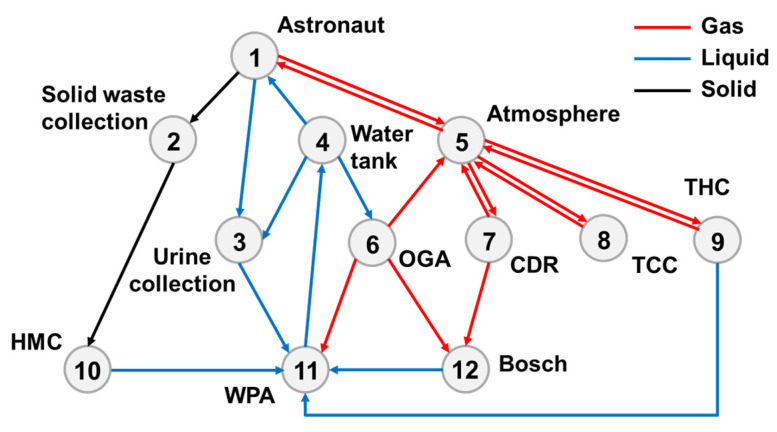
Directed network diagram of top-level scheme I.

**Figure 16:**
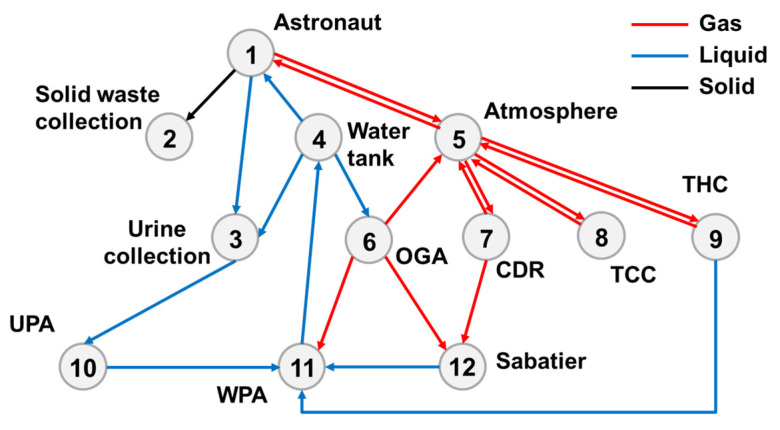
Directed network diagram of top-level scheme II/ISS.

**Figure 17:**
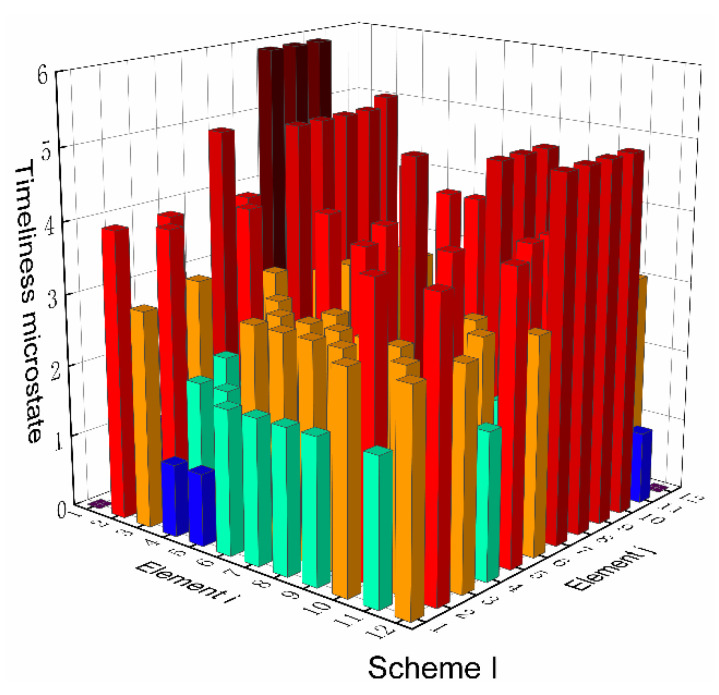
Timeliness microstate distribution of scheme I.

**Figure 18:**
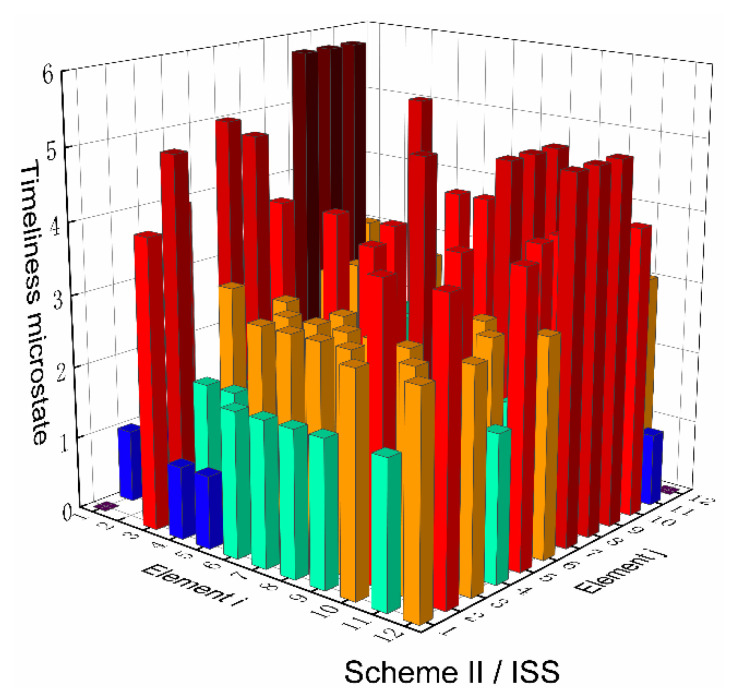
Timeliness microstate distribution of scheme II/ISS.

**Figure 19:**
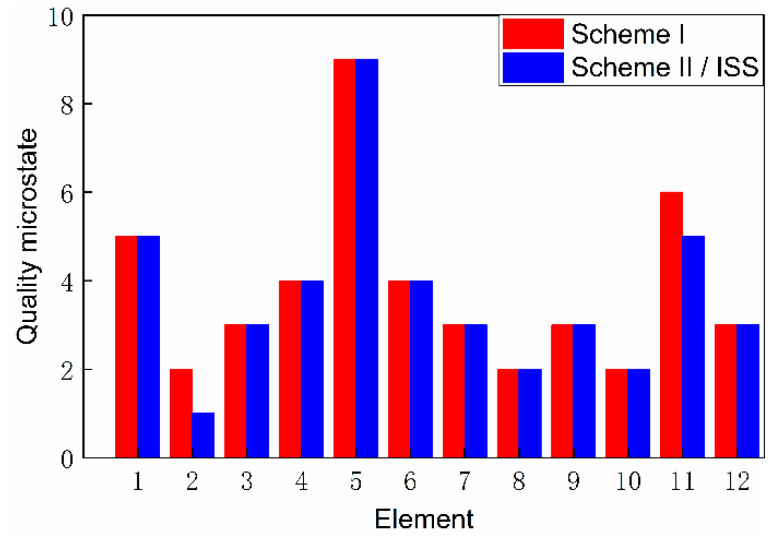
Quality microstate distribution.

Figure 15 and Figure 16 present the directed network diagrams of top-level scheme I, II and ISS. The timeliness microstates and quality microstates of each element are calculated based on the directed network diagram of the top-level scheme, as shown in Figure 17, Figure 18 and Figure 19.

**Figure 20:**
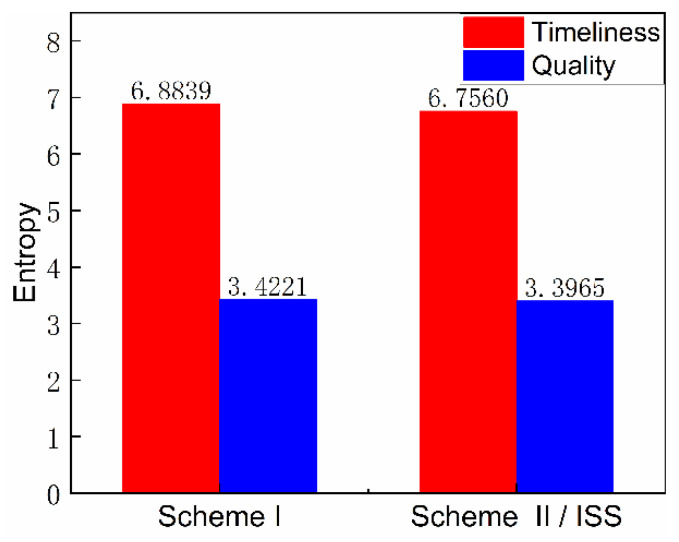
Timeliness and quality entropy.

**Figure 21:**
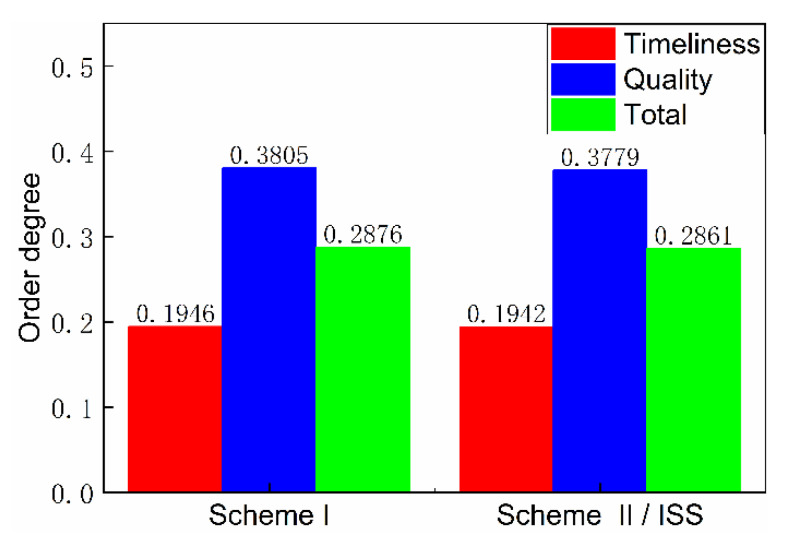
Order degree.

Figure 20 and Figure 21 illustrate the structure entropy and order degree of directed structure network diagrams. Considering the actual material flow direction of the system, the timeliness and quality entropy of scheme I are greater than those of scheme II/ISS, and the timeliness and quality order degree of scheme I are also higher than those of scheme II/ISS. This indicates that scheme I possesses better efficiency and accuracy of material flow than scheme II/ISS. Similarly, the total order degree is calculated by setting the weight of timeliness and quality order degree to 0.5, respectively. The total order degree of scheme I is slightly higher than that of scheme II/ISS.

According to the analysis of the D-SEM, the change of partial system structure and difference of material flow direction have little influence on the operation complexity of the system.

The above analysis preliminarily shows that U-SEM and D-SEM may be used to quantitatively describe system complexity. However, the current research only realizes the preliminary transformation from physical system to information system, wherein the process of information transmission and acceptance in the organizational framework of the system has been considered. The connotation of information cannot be reflected in the current algorithms. In further research, we hope to map the physical and chemical reactions or other thermal processes of ECLSS to the network structure. When the network structure can reflect the real physical system, it is possible to use the data of the actual system to verify the method. In the future research on ECLSSs, the complexity of system design may be more comprehensively evaluated.

## 5. Conclusions

This study focuses on the scheme design and the complexity evaluation of ECLSSs. Two schemes are designed based on the principle of improving system closure and reducing power consumption. The U-SEM and D-SEM are used to evaluate the complexity of the system. The results show that:

(1) According to the U-SEM and D-SEM, scheme I and II/ISS are nearly of equivalent complexity.

(2) The limited change in the system structure caused by partial technology replacement has little effect on the system complexity at the information level.

(3) The information transmission direction likely leads to some differences in the evaluation results of the system complexity.

These studies provide a calculation method of a system evaluation indicator for ECLSS top-level design. In future research, we will introduce actual physical system parameters to improve SEM and combine the information level and the physical level to evaluate system complexity.

## Figures and Tables

**Table 1:** Three life-support schemes.

Subsystem	Assembly	Scheme I	Scheme II	ISS [30]
Atmosphere revitalization	CO_2_ removal	4-bed molecular sieve	2-bed molecular sieve	4-bed molecular sieve
	CO_2_ reduction	Bosch	Sabatier	Sabatier
	Oxygen generation	Solid polymer water electrolysis	Solid polymer water electrolysis	Solid polymer water electrolysis
	Trace contaminant control	Adsorption + catalytic oxidation	Adsorption + catalytic oxidation	Adsorption + catalytic oxidation
	Temperature and humidity control	First generation condensation	Second generation condensation	First generation condensation
Water management	Water processing	Vapor phase catalytic ammonia removal	Multiple filtration + vapor compression distillation	Multiple filtration + vapor compression distillation
Waste management	Waste processing	Heat melt compactor	Collection compression	Collection compression

## Data Availability

Not applicable.

## Supplementary Materials

The following are available online at https://www.mdpi.com/article/10.3390/e23091173/s1, Supplementary Materials: Function introductions of each subsystem of ECLSS and performance index comparison of different technologies.

## Nomenclature

**Abbreviation**	
2BMS	two-bed molecular sieve
4BMS	four-bed molecular sieve
C	carbon
CC	collection and compression
CDR	carbon dioxide removal
CH_4_	methane
CO_2_	carbon dioxide
D-SEM	directed structure entropy method
ECLSS	environmental control and life-support system
FGC	first generation condensation
H_2_	hydrogen
HMC	heat melt compactor
H_2_O	water
ISS	international space station
MF	multiple filtration
O_2_	oxygen
OGA	oxygen generator assembly
SCM	system complexity metric
SEM	structure entropy method
SGC	second-generation condensation
TCC	trace contaminant control
THC	temperature and humidity control
UPA	urine processor assembly
U-SEM	undirected structure entropy method
VCD	vapor compression distillation
VPCAR	vapor phase catalytic ammonia removal
WFRD	wiped film rotating disk
WPA	water processing assembly
**Symbol**	
*H*	structure entropy
*K*	number of connections
*L*	minimum lengths
*N*	total number of microstates
*p*	realization probability of microstate
*R*	order degree
*α*	weight of timeliness
*β*	weight of quality
**Subscript**	
1	timeliness
2	quality
m	maximum
*i*	elements
*j*	elements
