# Supplementary material for: Complexity Evaluation of an Environmental Control and Life-Support System Based on Directed and Undirected Structural Entropy Methods
Source: Entropy (Basel). 2021 Sep 7;23(9):1173. doi: 10.3390/e23091173 (PMC8465968; doi:10.3390/e23091173)
Supplement: Supplementary file 1 [file entropy-23-01173-s001.zip › entropy-1329925-supplementary.pdf]

Supplementary materials

Complexity evaluation of the environmental control and life support system based on directed and undirected structural entropy methods

The following introduces several subsystems of ECLSS and the technical selection of each subsystem.

(1) CO_2 reduction

CO_2 reduction technology can enable the recovery of oxygen to improve the closure. At present, Bosch and Sabatier reduction methods are primarily applied. Particularly, the Sabatier reduction method with a relatively high technical maturity and good stability is applied to the ISS.

Sabatier reaction

$$CO_2 + 4H_2 \rightarrow CH_4 + 2H_2O \tag{1}$$

Bosch reaction

$$CO_2 + 2H_2 \rightarrow C + 2H_2O \tag{2}$$

The mass and power consumption of Sabatier reduction method are lower than that of Bosch reduction method, but the theoretical closure of Sabatier method is lower than that of Bosch reduction method, nearly 50%. Bosch reduction method can theoretically recover 100% oxygen from the CO_2 [1,2].

(2) Water management

The water management system makes physical and chemical processing of wastewater including urine and condensate water, and provides water for drinking, sanitation, oxygen production and urine flushing [3]. ISS pretreats urine firstly and then treats it together with wastewater using VCD. NASA is developing the physicochemical regeneration technology, which mainly includes the VPCAR and wiped film rotating disk (WFRD), without urine pretreatment [4]. Although the power consumption of VPCAR is higher, its water recovery rate is also higher than that of ISS [5-7].

(3) Waste management

At present, the waste collection system is only responsible for the collection and compression (CC) of waste, while the solid waste contains 25% moisture. Recycling this moisture can further improve the closure degree of the system. HMC is a waste treatment technology integrating water recovery, volume compression and treatment stabilization [8].

(4) CO_2 removal

In the manned space mission, it is necessary to remove CO_2 metabolized by astronauts in a certain way to ensure that the CO_2 concentration in the cabin remains within the acceptable range and avoid them any harm. ISS uses 4BMS, and the future development technology mainly includes 2BMS supported by hydrophobic adsorbents [9], as well as solid amine and ionic liquid adsorption [10]. The volume, mass and power consumption of 2BMS are lower than that of 4BMS, but the current technology maturity is not high.

(5) Temperature and humidity control

In the temperature and humidity control subsystem, the condensing and separating components directly determine the system performance. The existing condensing separation components mainly includes the FGC components composed of condensing heat exchanger, slurper structure, water vapor separator, etc. The SGC module integrates the functions of condensation heat transfer and water vapor separation [11]. The FGC components has high maturity, and the SGC has small volume and no rotating parts.

The OGA and TCC technologies of the two schemes are consistent with ISS. Solid polymer water electrolysis is used for oxygen regeneration [12]. Adsorption and catalytic oxidation is used for TCC [13,14].

Table 1 summarizes the performance comparison of different assembly, including recovery and power consumption.

Table 1. Comparison among the performance parameters.

**Assembly**	**Generation**	**Recovery**	**Power**
CO_2 reduction [1]	Bosch	<100 %	1.38 kW
	Sabatier	< 50 %	0.4 kW

Water management [5-7]	VPCAR	98 %	1.9 kW
	VCD	90-95 %	0.7 kW
Waste management	HMC	25 %	HMC > CC
	CC	--	
CO_2 removal	4BMS	--	4BMS > 2BMS
	2BMS	--	
Temperature and humidity control	FGC	--	FGC > SGC
	SGC	--	

In the future, ECLSS system will be developed in the direction of multiple functions or integration, with more significant reduction in mass and power consumption. For example, the new ionic liquid technology can provide an integrated system for removing carbon dioxide, humidity and trace contaminant [15]. As the maturity of these new technologies is limited, the technology selection of the overall scheme in this study will not be considered.

References:

1 Jones, H. Carbon dioxide reduction system trade studies. In Proceedings of the 41st International Conference on Environmental Systems, Portland, Oregon, USA, 17 - 21 July 2011. https://doi.org/10.2514/6.2011-5099.

2 Stromgren, C.; Escobar, F.; Anderson, M.S.; Stambaugh, I.; Sargusingh, M.J.; Goodliff, K.E. Assessment of Desired ECLSS Closure Rates for Human Mars Missions. In Proceedings of the AIAA SPACE and Astronautics Forum and Exposition, Orlando, FL, USA, 12 - 14 September 2017. https://doi.org/10.2514/6.2017-5123

3 Carter, D.L.; Tobias, B.; Orozco, N.Y. Status of ISS Water Management and Recovery. In Proceedings of the 43rd International Conference on Environmental Systems, Vail, CO, USA, 14-18 July 2013. https://doi.org/10.2514/6.2013-3509

4 Mackey, J.R.; Hall, N.R. Optical Thin Film Measurements using a Vapor Phase Catalytic Ammonia Removal System at Elevated Temperatures. In Proceedings of the

40th International Conference on Environmental Systems, Barcelona, Spain, 11 - 15 July 2010. https://doi.org/10.2514/6.2010-6099

5 Flynn, M.; Fisher, J.; Kliss, M.; Tleimat, B.; Tleimat, M.; Quinn, G.; Fort, J.; Nalette, T.; Baker, G.; Genovese, J. The Development of the Vapor Phase Catalytic Ammonia Removal (VPCAR) Engineering Development Unit. In Proceedings of the 34th International Conference on Environmental Systems, Colorado Springs, Colorado, USA, 19-22 July 2004.

6 Levri, J.A.; Vaccari, D.A.; Drysdale, A.E. Theory and Application of the Equivalent System Mass Metric. In Proceedings of the 30th International Conference on Environmental Systems, Toulouse, France, 10–13 July 2000.

7 Thompson, J.O.; Wheeler, A.R. Bead Evaporator for Complete Water and Salt Recovery from Brine. In Proceedings of the 45th International Conference on Environmental Systems, Bellevue, Washington, USA,12-16 July 2015.

8 Harris, L. C.; Wignarajah, K.; Alba, R.; et al. Characterization of Heat Melt Compactor (HMC) Product Water. In Proceedings of the 43rd International Conference on Environmental Systems, Vail, CO, USA, 14-18 July 2013. https://doi.org/10.2514/6.2013-3394

9 Wieland, P.O. Living together in space: the design and operation of the life support systems on the International Space Station. NASA, Washington, DC, USA, 1998 .

10 Nabity, J. A.; Killelea, J. V.; Shaffer, B. A.; Arquilla, K.S.; Rundle, T.J.; Fritz, T.J.; Phillips, D.D. Ionic-Liquid-Based Contactors for Carbon Dioxide Removal from Simulated Space-craft Cabin Atmospheres. *J. Spacecraft Rocke*t **2020**, 57, 1350-1361. https://doi.org/10.2514/1.A34750

11 Hasan, M.M.; Khan, L.; Nayagam, V.; et al. Conceptual design of a condensing heat exchanger for space systems using porous media. In Proceedings of the 35th International Conference on Environmental Systems, Rome, Italy, 11-14 July 2005. https://doi.org/10.4271/2005-01-2812

12 Carmo, M.; Fritz, D. L.; Mergel, J.; et al. Comprehensive review on PEM water electrolysis. *Int. J. Hydrog. Energy* 2013, 38, 4901-4934. https://doi.org/10.1016/j.ijhydene.2013.01.151

13 Perry, J.L.; Cole, H.E.; El-Lessy, H. An Assessment of the International Space Station's Trace Contaminant Control Subassembly Process Economics. NASA, Washington, DC, USA, 2005.

14 Macatangay, A.V.; Perry, J.L.; Belcher, P.L.; et al. Status of the International Space Station (ISS) trace contaminant control system. *SAE Int. J. Aerosp.* 2009, 4, 48-54. https://doi.org/10.4271/2009-01-2353

15 Yates, S. F.; Bershitsky, A.; Bonk, T.; Henson, P.; MacKnight, A. Direct Liquid Contact – Next Generation Approach to Combined CO2 Recovery and Humidity Control for Extended Missions. In Proceedings of the AIAA SPACE Forum, Long Beach, California, USA, 13 - 16 September 2016. https://doi.org/10.2514/6.2016-5462
